# Prevention of human exposure to livestock faecal waste in the household: a scoping study of interventions conducted in sub-Saharan Africa

**DOI:** 10.1186/s12889-023-16567-x

**Published:** 2023-08-24

**Authors:** Derrick N. Sentamu, Joseph Kungu, Michel Dione, Lian F. Thomas

**Affiliations:** 1 Animal and Human Health Program, International Livestock Research Institute, P.O Box 30709, Nairobi, 00100 Kenya; 2 College of Veterinary Medicine, Animal Resources and Biosecurity, Makerere University, P.O Box 7062, Kampala, Uganda; 3 International Livestock Research Institute, c/o AfricaRice, Rue 18 CitéMamelles, BP 24265, Dakar, Senegal; 4 Institute of Infection, Veterinary & Ecological Sciences, The University of Liverpool, Leahurst Campus, Neston, Liverpool, CH64 7TE UK

**Keywords:** Manure management, WASH, Zoonoses, Public health

## Abstract

**Background:**

Poorly managed animal faecal waste can result in detrimental environmental and public health implications. Limiting human exposure to animal waste through Animal inclusive Water Sanitation and Hygiene (A-WASH) strategies is imperative to improve public health in livestock keeping households but has received little attention to date. A small number of A-WASH interventions have previously been identified through a systematic review by another research team, and published in 2017. To inform intervention design with the most up-to-date information, a scoping study was conducted to map the existing evidence for A-WASH in sub-Saharan Africa (SSA) emerging since the previous review.

**Methods:**

This review followed PRISMA guidelines to identify interventions in SSA published between January 2016 to October 2022. Databases searched included PubMed, PMC Europe, CabDirect and Web of Science. Studies were eligible for inclusion if they were written in English and documented interventions limiting human contact with animal faecal material in the SSA context. Key data extracted included: the intervention itself, its target population, cost, measure of effectiveness, quantification of effect, assessment of success, acceptability and limitations. These data were synthesized into a narrative, structured around the intervention type.

**Findings:**

Eight eligible articles were identified. Interventions to reduce human exposure to animal faecal matter were conducted in combination with ‘standard’ human-centric WASH practices. Identified interventions included the management of human-animal co-habitation, educational programs and the creation of child-safe spaces. No novel A-WASH interventions were identified in this review, beyond those identified by the review in 2017. Randomised Controlled Trials (RCTs) were used to evaluate six of the eight identified interventions, but as effect was evaluated through various measures, the ability to formally compare efficacy of interventions is lacking.

**Conclusion:**

This study indicates that the number of A-WASH studies in SSA is increasing and the use of RCTs suggests a strong desire to create high-quality evidence within this field. There is a need for standardisation of effect measures to enable meta-analyses to be conducted to better understand intervention effectiveness. Evaluation of scalability and sustainability of interventions is still lacking in A – WASH research.

**Supplementary Information:**

The online version contains supplementary material available at 10.1186/s12889-023-16567-x.

## Background

More than half of global population growth between now and 2050 is expected to occur in Africa, with the population of Sub-Saharan Africa (SSA) projected to double by 2050 from 1.2 billion today [1, 2]. Population increase, alongside urbanisation and increasing disposable incomes, drive higher demand for livestock source food on the continent and in turn, increased domestic livestock production. Livestock production is one of the fastest-growing economic sectors in Africa and contributes between 30 – 80 percent of agricultural GDP across the continent [3]. This anticipated expansion of Africa’s livestock sector, if uncontrolled, has the potential for negative effects on public health, the environment and livelihoods, through the large amounts of manure produced by animals [4]. Through to 2030, Africa is projected to have the largest average annual faecal biomass change, estimated at 14 × 10^9^ kg/year. According to the Food and Agricultural Organization (FAO), domestic animals produce 85% of the world’s animal faecal waste, far greater than that produced by humans [5].

When not properly removed from human domestic environments, animal faeces expose humans to zoonotic pathogens through fecal–oral transmission from contaminated fingers, food and water sources [6]. Associations have been made between animal faecal exposure and human enteric infections, diarrhoea, stunted growth, poor cognitive development and even mortalities [7–13]. Little attention has been given to animal faecal pathogens transmitted via water, sanitation, and/or hygiene (WASH)-related pathways [14] and relatively few existing WASH programs currently strongly emphasize management of animal faeces [15].

A review by Penakalapati et al. [14] identified interventions implemented to reduce animal fecal contamination of humans and their environments. The search was global and included all interventions carried out before 3/10/2016. It returned seven articles with only one from Sub – Saharan Africa. Interventions described included reducing human-animal contact through the provision of chicken corrals and livestock fencing, creating safe child spaces by provision of play yards, clearing the household environment of animal faeces through the use of animal faecal scoops, where scooped animal faeces were discarded in provided household pit latrines, and promotion of hygiene through encouraging hand washing to avoid fecal contamination of food with hands.

Removing chickens from human homesteads had low uptake among households that didn’t house their poultry before the study [16]; in the intervention that provided corrals, it was observed that corralling might have perversely augmented the risk of campylobacteriosis in children [17]. For metal scoops used to remove animal feaces and dispose of them safely in a dual-pit latrine, the authors found it difficult to assess the effects of this activity alone [18]. For sani-scoops used for the disposal of child and animal faeces, relatively high usage of the hardware was reported, but no significant changes in observation of animal faecal matter around humans were observed pre and post intervention [19]. Improving animal veterinary care increased access to health services in most villages, reducing exposure to emerging infectious disease hazards. Educational activities under this intervention reduced cohabitation with livestock in one in three households [20].

In order to update the current evidence base on interventions to reduce human exposure to animal faeces at the household level in farming communities in Africa, this scoping review was conducted. It is a preliminary assessment of the size, scope, features, nature and conceptual boundaries of recent interventions in SSA. This review will also present the documented relevance to, adoption and acceptability by the target communities and populations in order to influence intervention design in ongoing and future projects. A scoping review approach was deemed a suitable approach given the need to identify and contextualise the current evidence prior to identifying more specific research questions relevant to a systematic literature review and meta-analysis approach [21].

## Methodology

A scoping review, guided by the Preferred Reporting Items for Systematic Reviews and Meta-Analyses (PRISMA) guidelines was conducted to update our understanding of Animal Inclusive WASH interventions published since the Penakalapati et al. [14] review. Manuscripts were identified from PubMed, Europe PMC, and CAB Direct databases, which detailed manure management practices to reduce human exposure to pathogens conducted in an African setting published between 1^st^ January 2016 and when the search was performed on the 13^th^ October 2022.

The syntax used for the four databases is in Additional File 1 with returned results as downloaded on the 13^th^ October are available in Additional File 2.

### Inclusion criteria

Any research article or report published between 1^st^ Jan 2016 to 13^th^ Oct 2022, which documented the application of an intervention to limit human exposure to livestock faecal waste in Africa, was considered.

### Exclusion criteria

Studies on pathogens in manure without an intervention described, studies on human faecal material management, studies on knowledge, attitudes & practices on manure management or studies documenting routine manure management without reference to limiting exposure of people to pathogens in manure were excluded from this review.

### Data extraction

Data extracted from the articles included; Title, Authors, Year, Location, Study design, Sample size, Target Livestock species, Target human population, The Intervention, Measure of Effect, Quantification of Effect, Cost, Author’s Assessment of Success, Acceptability and Limitations. These results were synthesized in brief narratives, grouped by intervention type. The narratives provide a description of the intervention, including the study location, target livestock species and human population (e.g., children or adults). A description of how the intervention was evaluated, including study design and sample size where appropriate, measure of effect, quantification of effect where appropriate, the author’s assessment of success and where reported, a description of the interventions cost, acceptability and limitations, was also included.

### Ethical approval

As this study identified and reviewed previously published literature no ethical approval was required for this scoping review.

## Results

**Fig. 1:**
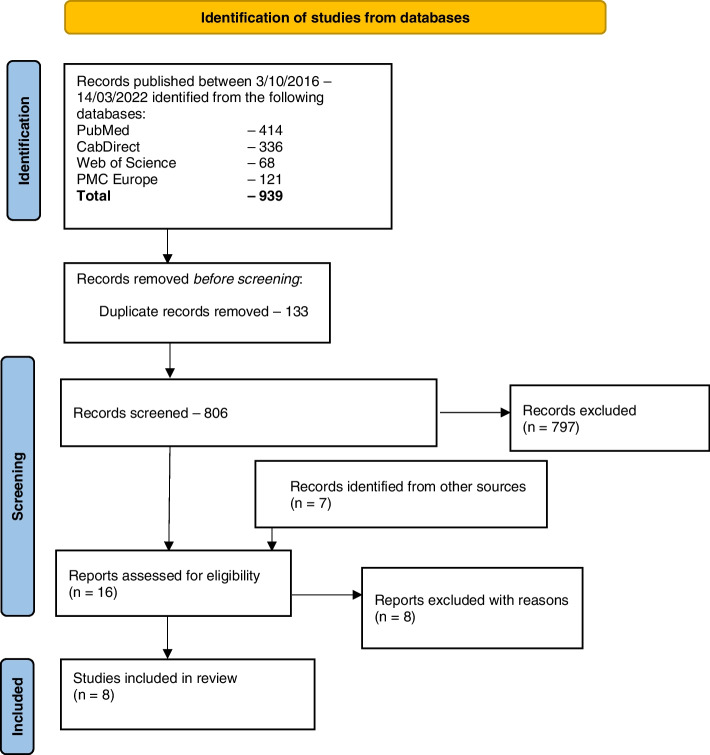
A flow chart of the literature search strategy

Figure 1 illustrates the screening process. Additional File 2 provides the list of all results returned from each database searched. Only eight articles met the inclusion criteria for data extraction which among them describe eight A-WASH interventions. In addition to these eight articles, eight more were published from these interventions; these were obtained from references cited in the included articles during full text screening. The eight interventions are summarised in Table 1, with all details from the data extraction provided in Additional File 3. Three of the studies originated from Ethiopia, while the others came from Zambia, Malawi, Kenya, Zimbabwe and the Democratic Republic of Congo (DRC). Six interventions were implemented using a randomised control trial study design, while non-randomised control trial and prospective cohort study designs were used for each of the other two studies. All animal faeces management interventions were designed as a component under larger ‘traditional’ human-centric WASH programs. One intervention focused on removal of chicken faeces, while all the other interventions were agnostic to the source of the animal faecal matter, focusing on reducing contact with faecal matter of any domestic animal species. Documentation of the cost of the intervention was only available for one intervention. For all studies, the primary aim of animal faecal matter removal was to safeguard the health of children, whose ages ranged from one month to five years.

**Table 1:** A summary of the results

**Authors**	**Country of Study**	**Intervention Category**	**Evaluation Study design**	**Measure of effect**	**Length of follow-up**	**Outcome of intervention**
[22]	Zambia	Child-safe spaces (explain in one sentence)	Randomised Control Trial	Self-reported use of play-yard Observation of faeces in the play-yard Qualitative assessment of benefits and usability	1 month	Reported usage of play yard Children protected from mouthing animal faeces
[23] Other Publications [24–26]	Malawi;	Education Intervention	Cluster Randomised Control Trial	Self-reports for diarrhea and respiratory infections Microbial tests for the same infections	9 months	Significant reduction in diarrhea Significant reduction in respiratory infections
[27] Other publications [28, 29]	Kenya	Education intervention	Cluster Randomised Control Trial	Observation of number of households without faeces in the compound	5 months	Marked increase in number of households without animal faeces in compound in the intervention arm
[30]	Ethiopia	Creation of child safe space	Randomised Control Trial	E. coli microbial load tests Observation of number of interactions with the playpen Self-reports regarding usage of the playpens	1 month	Significant difference in E. coli contamination levels of playpen and house floors
[31] Other publications [32]	The Democratic Republic of Congo	Managing cohabitation with animals	Prospective cohort study	Measurement of children HAZ scores Children caregiver reports of touching animals Researchers’ observation of animal faeces in children sleeping spaces	6 months	Marked reduction in number of children mouthing animal faeces
[33] Other publications [34]	Zimbabwe	Creation of child safe space	Cluster Randomized control trial	Microbial tests	12 months	Decreased prevalence of total enteric parasites Reduction was not significant
[35]	Ethiopia	Managing cohabitation with animals	Cluster randomized trial	Researchers’ observation of animal faeces	Not reported	Increase in number of households without animal faeces
[36] Other publications [37]	Ethiopia	Creation of child safe space	Randomised Control Trial	Qualitative based on observation and reports; Quantitative based on microbial load measurements	1 month	Reduced usage of the HPS by an average of 1 h over 1 month Significant reduction in prevalence of diarrhea No significant reduction in Campylobacter load

Four interventions focused on the use of child-safe spaces [22, 30, 33, 36], two interventions focused on managing cohabitation between humans and animals by encouraging building of animal hutches/coops [31, 35] and two educational interventions encouraged appropriate removal of faeces from the household environment [23, 27].

The measures of effect of the interventions were varied but included: use of microbial tests (*n* = 4), change in Height for Age Z (HAZ) scores (*n* = 1) and the observation or self-report of the presence of animal faecal matter around children, cleanliness of children’s hands and change in a behaviour of interest.

The next section of the results provides a narrative description of the interventions under the data extraction items. The categories of interventions are highlighted by heading. The different studies are reported and described by paragraph.

### Managing cohabitation with animals

Two studies focussed on managing the cohabitation of livestock with humans. Passarelli et al. [35] conducted a study in Ethiopia using a cluster randomised trial, dubbed “Agriculture-to-Nutrition” (ATONU). The objective of the larger study was to evaluate the impact of integrating nutrition-sensitive behavioural change communication in the context of increased household production of chickens and eggs on the diets of women and children. This study targeted children from 0 – 36 months. The intervention arm received good breeds of chicken and behaviour change communication. Households in the intervention arm were encouraged to use local materials to construct a chicken coop. Three types of coops were observed, categorised according to 1) being open or closed (entry), 2) distance from the house. The effect of this intervention was based on observation of chicken faeces around the household by enumerators and cleanliness of children’s hands. The findings indicated that having any kind of chicken coop significantly increased the risk of animal faeces in the compound by 30% and was not associated with child cleanliness. For households with chicken coops, if the coop was closed (relative to being open), there was increased likelihood of no faeces being observed and children’s hands being clean. All types of chicken coops besides the open housing, significantly improved the likelihood of the index child having clean hands. A chicken house located 1 m away from the household (compared to being inside or attached to the house) was also positively associated with the two outcome measures of cleanliness. Lack of feed for the chickens was identified as the major constraint against utilization of chicken coops. The average cost of this intervention was undocumented.

A prospective cohort study in DRC by Kuhl et al. [31] provided animal hutches made of locally available materials (rags, wood, and bamboo) to house rabbits, guinea pigs, ducks, and chickens. Participants strongly resisted building hutches outside due to fears of animal theft or death from the cold, hence they were built indoors, mimicking the design found in the community. Hutches had three levels where the top one housed rabbits, the second kept guinea pigs, and the bottom one housed chickens, turkeys, and ducks. Rabbits and guinea pigs were kept in the hutch 24 h a day. Participants were highly encouraged to sweep up animal faeces from the household compound and disposing of it far away from children’s play areas. They were also advised to use locally available materials to construct a compost pile that would enable use of animal excrement in combination with other biodegradable organic waste. The target population for this intervention was children under five years. Therefore, to measure its effect, HAZ scores after a six-month period were used. There was a significant reduction in HAZ scores and caregivers self-reported reductions in children’s contact with animals and mouthing of animal faeces [32]. The cost of this intervention was undocumented. In the end, the intervention was rolled out wider to over a million participants.

### Education interventions

Some interventions focused entirely on education and increasing awareness. An intervention framework for a cluster randomised trial targeting children under five years in Malawi by Morse et al. [23] was based on the critical points that were informed by a prior formative research. Four thematic areas, including animal faeces management, were examined in terms of context (social, personal, and environmental), structural barriers, and psychosocial factors to design specific intervention activities primarily targeting behavioural change. These activities were developed with an in-house design team to produce specific and complementary modules, deliverable through community-based volunteers with support from community health workers. The education intervention package concerning the management of animal faeces involved; creating disgust through a faeces-eating game using water and food; employing posters, videos, games, plays and role-playing to demonstrate appropriate hygiene and faecal removal processes. The intervention encouraged local administrative commitment and peer-to-peer knowledge exchange to increase uptake of desired behaviours, along with a reward system for good practice performers. Analysis of self-reports for diarrhoea and respiratory infections showed: at baseline, the treatment and control values for diarrhoea and respiratory infections did not significantly differ, but by endline, there were significant differences between each treatment area and the control area for diarrhoea. For respiratory infections, the difference was only between one of the treatments and the control [24–26]. The intervention cost towards the faeces management theme was $1191, where the cost per household was $4.77. This was exclusive of costs incurred in the development of the intervention, but included only those relating to training of community coordinators and delivery of the intervention to the population. Limitations from this study included: unmeasured male participation at household visits, thus strategies to encourage their participation could not be obtained; study duration limited to nine months; not all variables that may affect the prevalence of diarrhoea were measured; a small sample size; and the use of the number of attendees to measure reach of the intervention.

Another study in Kenya, targeting children two years and less by Wodnik et al. [27], designed a demand-side integrated intervention to improve nutrition and WASH behaviours. The study focused on teaching a series of key messages about WASH behaviours. Caregiver behavioural change, in keeping the children’s environment free of animal faeces, was targeted. Training of the participants included demonstrations on how to scoop faeces from the ground using a faeces scooper, disposing of it in the latrine, sweeping the compound to remove animal faeces, and information on the links between animal faeces and ill child health. Participants were also encouraged to buy a faeces scooper. There was a significant increase in the number of people with an animal faeces free environment in the intervention arm (15 at baseline and 40 at endline), but no change in the control arm (23 at baseline and 24 at endline) [28, 29]. This study was limited by a short length of follow up, small sample size and lack of health-outcome data evaluated.

### Creation of child safe spaces

Most interventions involved the development or use of a play yard to separate children from animal faeces. Reid et al. [22] carried out a randomised control trial intervention targeting young children between six and 24 months. This intervention compared two groups provided with different designs of play yards. After delivering a Baby WASH education module, leaders and community members in the intervention group were engaged in a participatory co- design session. A research team with expertise in design, child development, and international nutrition developed the basic design requirements of the community-built Baby WASH play-yard. Through an interactive process, a play yard made of local materials was created and adopted. For the control group, a plastic play-yard manufactured in the United States was distributed. Both play yards were delivered after education modules. Three assessment visits involving a short interview and observation of use of the play yard and the environment followed. The impact of the play yards was assessed based on self-reports and observations of the frequency of use of the play yard.

Both groups reported using the play-yard mostly in the morning and afternoon, with the duration of use episodes ranging from 10 min to three hours. Regarding cleanliness, no animal faeces were observed within the yard at any point. The presence of dirty toys and insufficient adult supervision were the most common hazards in the community play yard group while visible dirtiness and risky placement of the play-yard, such as near an open wall or fire pit, were common hazards observed in the plastic play yard group. Limitations of this study included: a small sample size; short study term; lack of refined qualitative assessments of community acceptance; the study was conducted during a dry season, so it needs to be conducted during a wet season to observe the utilisation of the intervention, reliance on self-reports and 24 h recalls; limited observation time by the research team; which arises from the possibility that caregivers changed their baby WASH related behaviours due to presence of an observer.

Another intervention in Zimbabwe targeting children of one and 18 months [33, 34] explored the use of a washable, locally manufactured mat and plastic play yard provided at two and six months respectively. There was a decrease in the prevalence of total parasites, but not bacterial or viral infections. Intervention impacts for individual pathogens, for both enteric infections and pathogen-attributable diarrhoea, were small and not significant. Limitations from this study included relatively few time points sampled to capture enteric infections.

An intervention by Rosenbaum et al. [30] in Ethiopia targeted children aged between seven and 12 months. It trialled three playpen types with different designs; Model A was imported, Model B and C were locally designed but different. The playpens were distributed and follow up visits made involving qualitative data collection using questionnaires and microbial sampling of the common room and the playmat within the playpen. E. coli was detected on 78% of the playmats, albeit at densities profoundly lower than those on the floors. No relationship was detected between the type of playpens and the contamination level. Considering all three playpen models, the mean reported amount of time infants spent in a playpen was 134 min per day at Visit 2 (SD = 100 min), and 123 min per day at Visit 3 (SD = 84 min). Limitations reported from this study included only short-term use, acceptability and feasibility of the playpens was examined. Time spent on dirt floors by study and non-study infants was not documented. Little is known about the thresholds of animal excreta and contaminated soil, and their effects on health and child growth. The study could not conclusively state that the comparative time children spent on cleaner surfaces of the playpens resulted in lower exposure to pathogens, leading to measurable health or growth benefits. The limitations included a small sample size, short study duration and limited generalisability. The relative brief period of use in households also restricted the accuracy of use reports as the infants aged. The cost of this intervention was not documented.

Budge et al. [37] conducted a study in Ethiopia, targeting infants aged 8 to 16 months from low income contexts, through a randomised control trial. This study designed and tested the acceptability of a House Play Space (HPS). A multisectoral participatory process engaged local Ethiopian stakeholders, while the design of the HPS occurred in the UK. The outputs from these two activities was presented in a workshop with local manufacturers to tailor the design to local needs. As a result, a foldable HPS was produced from local materials, meeting ISO safety standards. A trial was then designed and distributed to assess acceptability. Qualitative assessment of the HPS impact, based on observation and reports, showed that all households in the trial were using the HPS on the first unannounced visit. Quantitative assessment, based on microbial load, showed a notable reduction in the seven-day diarrhoea point prevalence in the intervention group, from 19 cases at the start to five cases at four weeks. In contrast, the control group saw a decrease from 22 cases to 16. The presumptive prevalence of Campylobacter remained high. However, from the baseline, the point prevalence showed no significant difference between the groups or time points. There was no decrease in the odds of Campylobacter-positive stool in any of the groups from the baseline. Overall, the HPS showed mixed engagement and adherence but had good acceptability among the study households that received an HPS. The acceptability of the HPS use, design and time use were reported [36]. The study’s limitations included a small sample size, a short timeframe, issues with the data quality and the reliance on self-reported data, which typically hold intrinsic inaccuracies. The study did not assess economic demand, HPS cost, or household willingness to pay. It was challenging to develop an affordable HPS option, which limits the intervention’s scalability.

## Discussion

Our search found a limited number of articles, corroborating the long-standing observation of the non-integration of the animal component in WASH interventions. It’s hypothesised that this may be due to the under-recognition of the impact of animals to human health [15, 38]. The scantiness of existing interventions could be attributable to the difficulty of utilising or implementing robust designs which are often expensive. Similar reports from Jimenez et al. [39] are attributed to associated contextual challenges for example accessibility of location, funding availability, ease of recruitment, political and social barriers. Collectively, the above postulations partly explain the stagnation in creativity concerning the separation of animal waste from humans, given our search yielded no new type of intervention compared to Penakalapati et al. [14]. Nevertheless, this search, having retrieved eight interventions over a seven-year period, compared to Penakalapati et al. [14], who obtained only nine articles from a much longer search period (all digitally available literature before 2016) and wider geographical range, suggests a relative increase in consideration of the health impacts of animals on human health. Furthermore, Penakalapati et al. [14] only identified one intervention in SSA [33], indicating a significant increase in interventions conducted over the last seven years.

Most interventions (*n* = 6) utilised the Randomised Controlled Trial (RCT) study design for their evaluation. Given their comparative nature and strong ability to make causal inferences, RCTs are the hallmark of evidence – based trials. Additional advantages of using RCTs include minimisation of allocation and selection bias due to randomisation [40]. Other advantages related to this design include minimisation of assessment and performance bias through double blinding [36] and the ability to observe indirect effects. Some studies went further to use Cluster Randomised Control Trials (CRTs) where interventions were applied to whole communities [23, 33, 35]. However, several limitations were identified that hindered the extrapolatory power of the evaluations, including small sample sizes, inability to fully address confounding factors, short duration of the interventions, seasonality and recall bias. Most of these challenges could be due to the costly nature of these types of studies [40].

A key limitation identified, which hinders the formal evaluation of intervention efficacy, is the variety of measures of effect employed. Whilst some interventions were assessed based on their direct impact on microbial load [23, 30, 33, 36], others used growth-based outcomes [31] and still others used proxy indicators. Proxy indicators are substitutes for common context indicators, such as relying on the observation of children’s hand cleanliness as an indicator for the presence of animal faecal pathogens, rather than directly measuring microbial load. These concerns are validated by the general consensus of the risk of attribution bias that arises from the variability around the actual relationship between the two indicators [41]. The diversity of outcome measures limits our ability to formally compare the efficacy of different interventions.

Acceptability is often central to WASH interventions. Various assessment measures, as described by Hosking et al. [42] can be used to measure acceptability of an intervention. Articles identified in this search utilised validated behavioural frameworks including Trials of Improved Practices (TIPs) used by [30, 36], a combination of TIPs and the COM – B framework [27], the Risks, Attitudes, Norms, Abilities, and Self-Regulation model [23] and the Integrated Behavioural Model (IBM) for WASH [31]. Acceptability from the use of these models was observed post-intervention, based on positive self-reports, observations of behavioural changes and reductions in pathogens and infections resulting from the interventions. Reid et al. [22] ensured community acceptability by engaging the community in the development process of the intervention, a community play yard, which was readily adopted by the participants. The remaining studies made no mention of acceptability. In all but one article, the assessment of acceptability was the end point of the intervention, with no inherent plan for evaluation of scalability or sustainability within the design of the intervention as recommended by UNICEF [43] in its 2016 – 2030 strategy for WASH programmes. Scalability can be defined as an estimation of the number of people that have received the intended ‘effect’ of the programme over time during and after the project’s implementation. Sustainability, on the other hand, is a function of scalability and cost of the intervention [44]. Sustainability considers financial, environmental, institutional and socio-cultural aspects, in addition to the technical intervention [45]. Without reporting the assessment method, only Kuhl et al.  [31] reported scalability to a wider population of over 1 million, while the cost of the intervention was stated by Morse et al. [23] without any mention of scalability or sustainability. Budge et al. [36] acknowledged that, despite the intervention undertaking an iterative process with stakeholders and utilising locally sourced materials, the cost of the technology would still pose a constraint to scalability. Similar findings on these concepts are reported by Jimenez et al. [39].

Despite the relative increase in the number of interventions in SSA, the general need for more effective animal-inclusive transformative WASH interventions persists. The demand side of the technology should be considered during its development. Animal faecal pathogens play a well known role in the contamination of water and water sources; there is a need to explore how this can be prevented. We observed that interventions mainly targeted children five years and below, at both household and community levels. No observable differences were able to be determined between age groups of children due to differences in age-groupings and lack of age-disaggregated data within cohorts. Age disaggregation even within cohort of ‘children under 5yrs’ would be important given the rapid development of children and potential requirement for unique approaches targeted at each developmental stage. This review highlights the need to broaden the settings and contexts in which animal faeces and faecal contamination can be reduced. For example, interventions could be more inclusive of youths and adults while disaggregating by sex. Other settings can also be explored, such as animal and human markets, slaughterhouses among others. Specific focus should be given to the different husbandry systems in Africa. We believe that A-WASH related interventions should not stand alone; they should be linked to livelihood or environmental sustainability outcomes. For instance, the management of animal waste through composting could improve crop production and increase household income. We recommend that future interventions actively seek for political commitment. This involves robust and diligent engagement of legislators and policymakers in the intervention design, implementation and communication of findings, for evidence-based policy making. Policy review should be explored to identify policies directed towards the management of animal manure, followed by evaluation of their implementation and effects and the creation of concise policy briefs on recommendations.

Lastly, there is a need to develop an evidence base around the operationalisation of a One Health approach in the implementation of A – WASH interventions in sub-Saharan Africa. One Health recognises the close link and interconnectedness between human, animal and the environmental health. One Health is inherently transdisciplinary and would draw on the strengths of multisectoral collaboration among all relevant disciplines to realise success in interventions [46]. Among other operational approaches, The World Bank [47] provides a clear guide for applying the One Health operational framework in project phases. This can be customised and tailored to A – WASH interventions.

This review should be interpreted in light of some limitations. A limited number of online databases were searched, and we did not include searches from grey literature or languages other than English. More information on A-WASH programmes could exist in these sources. A somewhat basic scoping study design was chosen due to the exploratory and broad nature of our research aim. Therefore, we included all interventions relevant to reducing human contact with animal faeces without applying inclusion criteria based on methodological rigour or specific measures of outcome effect. This review considered interventions that focused on removal/reduction of human microbial contamination through animal faecal contact. Interventions looking at other forms of public health hazards linked to environmental contamination by animal faeces, for example, antimicrobial residues and heavy metals, among other contaminants, were not considered.

## Conclusion

Owing to the absence of a comprehensive exploration of the body work in this field, this scoping review provides a preliminary consolidation of all recent A – WASH interventions in sub-Saharan Africa. Although this study is not entirely novel, building as it does upon Penakalapati et al. [14], this exploratory work is necessary for a nascent topic for which the evidence base is growing, but where standardised guidelines for evaluation and reporting of interventional trials have not been developed. The interventions identified in this review include managing cohabitation of humans with animals, creating of child-safe spaces and educational interventions to promote the cleaning of household environments. These specific practices can all be carried out in combination with other traditional human-centric WASH practices. The review highlighted a relative increase in the number of A-WASH interventions in SSA in the last seven years, representing a positive trend with stronger evidence for replicability of similar studies. More A – WASH interventions are encouraged, and their value could be improved through a standardisation of effectiveness measures to demonstrate quantitative impact on human health and to allow comparison between interventions. Quantitative measures should continue to be complemented with qualitative studies on acceptability and feasibility of the interventions trialled and assessment of the relative costs of interventions to enhance ability of communities to choose the most appropriate solutions for their context.

### Supplementary Information

**Additional file 1.**

**Additional file 2.**

**Additional file 3.**

## Data Availability

All data generated or analysed during this study are included in this published article.

## Abbreviations

(A-)WASH: Animal Inclusive Water Sanitation and Hygiene,
DRC: Democratic Republic of Congo,
HAZ: Height for Age Z score
HPS: House Play Space
PRISMA: Preferred Reporting Items for Systematic Reviews and Meta-Analyses
RCT: Randomised Controlled Trial,
SSA: Sub-Saharan Africa
