# Supplementary material for: Prevention of human exposure to livestock faecal waste in the household: a scoping study of interventions conducted in sub-Saharan Africa
Source: BMC Public Health. 2023 Aug 24;23:1613. doi: 10.1186/s12889-023-16567-x (PMC10463677; doi:10.1186/s12889-023-16567-x)
Supplement: Supplementary file 1 — Additional file 1. [file 12889_2023_16567_MOESM1_ESM.docx]

The search syntax used for the four databases.

**PubMed https://pubmed.ncbi.nlm.nih.gov/advanced/ (returned 414 results)**

(Animal OR Livestock OR sheep OR goat OR cattle OR pig OR Chicken OR Poultry OR Camel) AND (feces OR fecal OR waste OR manure OR dung OR droppings) AND (Disposal OR control OR management OR containment OR treatment OR intervention OR reduction) AND (Pathogen or Microbe) AND (Africa OR Angola OR Benin OR Botswana OR Burundi OR Cameroon OR Cape Verde OR Central African Republic OR Congo OR DRC OR Comoros OR Côte d'Ivoire OR Djibouti OR Equatorial Guinea OR Eritrea OR Ethiopia OR Gabon OR Gambia OR Ghana OR Guinea OR Guinea-Bissau OR Kenya OR Lesotho OR Liberia OR Madagascar OR Malawi OR Mali OR Mauritius OR Mozambique OR Namibia OR Niger OR Nigeria OR Réunion OR Rwanda OR "Sao Tome and Principe" OR Senegal OR Seychelles OR Sierra Leone OR Somalia OR Puntland OR Somaliland OR South Africa OR Sudan OR Swaziland OR Tanzania OR Zanzibar OR Togo OR Uganda OR Sahara OR Zambia OR Zimbabwe OR "east Africa" or "horn of Africa" or "southern Africa" or "west Africa" or "central Africa" or "southern and eastern Africa" or "sub-Saharan Africa") Filters applied: *from* *2016/1/1 - 2022/10/13*

**PMC Europe (returned 121 results)**

**https://europepmc.org/**

(“Animal” OR “Livestock” AND “Manure” AND “Disposal” AND “Pathogen” AND “Africa”) AND (FIRST PDATE:[2016 TO 2022])

**Web of Science (returned 68 results)**

**https://www.webofscience.com/wos/woscc/advanced-search**

((((ALL= (Animal OR Livestock OR sheep OR goat OR cattle OR pig OR Chicken OR Poultry OR Camel)) AND ALL= (faeces OR fecal OR waste OR manure OR dung OR droppings)) AND ALL= (Disposal OR control OR management OR containment OR treatment OR intervention OR reduction)) AND ALL= (Pathogen or Microbe)) AND ALL=Africa

Filter by publication date 2016-01-01 to 2022-10-13

**CAB Direct (returned 336 results)**

**https://www.cabdirect.org/cabdirect/search/**

(Animal OR Livestock OR sheep OR goat OR cattle OR pig OR Chicken OR Poultry OR Camel) AND (faeces OR fecal OR waste OR manure OR dung OR droppings) AND (disposal OR control OR management OR containment OR treatment OR intervention OR reduction) AND (Pathogen OR Microbe) AND (Africa) AND yr: [2016 TO 2022]
